# The value of one versus three sputum smear examinations for diagnosis of pulmonary tuberculosis in Asella hospital, South-East Ethiopia

**DOI:** 10.1186/s13104-017-2797-0

**Published:** 2017-09-06

**Authors:** Fekadu Beyene

**Affiliations:** Department of Internal Medicine, College of Health Sciences, Arsi University, Asella, Ethiopia

**Keywords:** Pulmonary tuberculosis, Sputum smear, Ethiopia

## Abstract

**Objective:**

The aim of the study is to compare the value of a single with three sputum smear examinations in the detection of smear-positive pulmonary tuberculosis.

**Results:**

There were a total of 7012 patients studied out of which 3599 (51.3%) were males and the rest females. In 637 (9.1%) of the patients, two or more smears were positive for AFB. 616 (96.7%) of the sputum smear positive patients had positive smears on the first spot sputum exams as compared to 635 (99.7%) who were positive on the morning sputum, (P = 0.000064). 598/637 (93.9%) of sputum smear positive patients had positive smears in all three smears regardless of the smear grading. A single morning smear examination is as sensitive as doing three sputum smear examinations in the diagnosis of sputum smear positive TB. The incidence of sputum smear positivity differed significantly across age groups, but did not differ between genders.

**Electronic supplementary material:**

The online version of this article (doi:10.1186/s13104-017-2797-0) contains supplementary material, which is available to authorized users.

## Introduction

Tuberculosis is a major global health problem causing ill-health among millions of people each year and ranks alongside the human immunodeficiency virus (HIV) as a leading cause of death worldwide. Ethiopia is one of the 22 high burden countries with incidence (including TB + HIV) of 207/100,000 population [1]. Pulmonary tuberculosis (PTB) has been of public health concern because sputum smear positive patients are the most infectious and most likely to transmit their dis-ease in their surroundings; they are the focus for infection control measures [2]. The diagnosis rests mainly on isolation of the causative organism from the appropriate specimen (sputum, lymph nodes, body fluids, etc.) by direct microscopy or culture. Recent advancement and technology in field has made it possible to make the diagnosis by serological, immunological and gene analysis [3–7]. But these tests have a limited sensitivity and/or specificity for detection of *Mycobacterium tuberculosis* (*M tuberculosis*), or are inaccessible to the developing world where the disease burden is at the climax, due to economic and technological limitations. International union against tuberculosis and lung diseases (IUALD), the World Health Organization (WHO), and the Federal Democratic Republic of Ethiopia (FDRE) national tuberculosis control program prepared guidelines recommend three sputum specimens; i.e. spot-morning-spot for diagnosis of new PTB cases [8–11].

The diagnosis of PTB is made if any two of the three sputum smears are positive for Acid Fast Bacilli (AFB) and is graded from 0 to 3+ [9].

To submit three sputum samples, patients need to stay for at least 2 days in the hospital. This has significant influence on the patients in terms of time and money they spend and leads to some patient loss. Processing multiple sputum smears leads to increased workload for the laboratory staffs and incurs additional cost for the health institution. If examining a single sputum smear sample gives the same result as examining three sputum smear samples without missing the smear positive TB patients, it would be advantageous for the patients, the laboratory personnel and the hospital for efficient resource utilization. Therefore, this study aims to know the overall sputum AFB positivity rate in the study population, to see the yield of sputum smear positivity in the three sputum samples, to see the difference in the sputum smear grade among the three samples, and to know the age group most affected by smear positive pulmonary TB.

## Main text

### Methods

A retrospective, cross-sectional study was done in Asella hospital, on laboratory records of all sputum smear examinations done for suspected pulmonary TB patients between May 01, 2014 and September 30, 2015. Asella hospital is 175 KM South-East of the Capital, Addis Ababa. It is one of the regional referral hospitals, serving for estimated 3.5 million people.

Records of sputum smear done in Asella hospital during the study period for suspected TB patients above the age of 15, were retrieved. Sputum smear results with incomplete bio-data and for those who are already on anti-TB treatment were excluded from the study. The data were entered to a prepared format on SPSS software (version 21.0), consisting of patients’, age, gender, sputum AFB status, sputum AFB for spot, morning and spot exam grading using Zhiel-Neelson method according to WHO and IULD recommended protocol [8, 9]. The technique consists of making a smear and drying it for 15–30 min, fixing it over the flame and staining with steaming hot carbol fuchsin for 5 min, decolorisation with 25% sulphuric acid for 2–4 min and counterstaining with 0.1% methylene blue for half a minute and examining under oil immersion lens of microscope. The number of positive smears of the first spot (the first spot sputum collected as the patient appears to laboratory), morning (the next day morning sample before having breakfast) and the second spot smears spot (the next day spot sample while the patient submits the morning sample) were compared with each other and with the two positive smears of the national standard to initiate anti-TB treatment. Continuous variables were described as mean (±standard deviation). Categorical variables were described as proportion and were analyzed to compare the significance of difference in distribution by using Chi square test. The difference in distribution was considered significant if *p*-value was less than 0.05.

## Results

There were a total of 7012 patients who fulfilled the inclusion criterion out of which 3599 (51.3%) were males.. In 637 (9.1%) of the patients, two or more smears were positive for AFB fulfilling the national standard criterion to initiate anti-tuberculosis (anti-TB) medications.

Six Hundred Sixteen of the sputum smear positive patients had positive smears on the first spot sputum smears (96.7%) as compared to 635 (99.7%) on the morning sputum (*P* = 0.000064) (Table 1).

**Table 1:** Contribution of sputum smear positivity by the three sputum smears (The italic numbers indicate the sensitivity of that single sputum as compared to the standard two positive smears)

AFB status		Spot 1		Morning		Spot 2	
		Positive	Negative	Positive	Negative	Positive	Negative
Positive (n = 637)	Count	616	21	635	2	618	19
	%	*96.7*	3.3	*99.7*	0.3	*97*	3
Negative (n = 6375)	Count	2	6373	3	6372	4	6371
	%	0	100	0	100	0.1	99.9
Total (n = 7012)	Count	618	6394	638	6374	622	6390

One Hundred Fifty-Two (23.9%) of sputum samples in spot 1 and 152 (24.8%) in spot 2 had high smear grades (i.e., 3+), while they were 228 (35.8%) in morning sputum smear (*P* = 0.00001) (Additional file 1: Figure 1). Five Hundred Ninety-Eight (93.9%) of sputum smear positive patients had positive smears in all three smears regardless of the smear grading (Table 2); 44.3% of them had the same sputum smear grading (Additional file 1: Figure 2).

**Table 2:** The three smears cross tabulations

	Morning (+)	Morning (−)
Spot 1 (+)/spot 2 (+)	598	1
Spot 1 (+)/spot 2 (−)	18	1
Spot 1 (−)/spot 2 (−)	2	6369
Spot 1 (−)/spot 2 (+)	20	3

There is significant difference in sputum smear positivity among age groups: 303 (15.3%) of all smears done are positive in the 15–25 age group as compared to 256 (8.8%) in 26–45 (*P* < 0.00001) (Table 3).

**Table 3:** age category v AFB status

Age category	AFB status		% of positive	
	Positive	Negative		
15–25	303	1682	4.3	
26–45	256	2664	3.7	*P* < 0.00001
46–60	59	1321	0.8	
>60	19	708	0.3	
Total	637	6375	9.1	

There was no difference in smear positivity between genders: 346 in males and 291 in females (P = 0.113).

## Discussion

The sensitivity of sputum microscopy has been reported to vary 20–80%, often depending on different factors [12]. The smear positivity among TB suspects in this study population was 9.1%, higher than that reported from study done in Tanzania tertiary hospital (6.1%) [13], but much less than that done in Rwanda (17.3%) [14], Turkey 42% [15] and Pakistan (52%), [16] where three consecutive morning sputum samples were examined. As the Bugando Medical Center in Northern Tanzania is a referral, consultant and teaching hospital, it may have contributed to the apparently lower yield of AFB in this patient population. The higher yield of smear positivity in later studies may be due to the high yield of AFB from the morning sputum sample. A one and half-year study done in India showed a smear positivity rate of 13.1% [17]. Another study from the same country for 2 years showed a smear positivity of 19.2% in 1998 and 24.9% in 1999 [18]. A more recent study from India showed a smear positivity rate of 16% with a positivity rate of 98.3, 98.6, and 96.9% for spot 1, morning, and both sputum smear examinations, respectively [19]. This is similar to the finding in our study: spot 1, morning and spot 2 showing 96.7, 99.7 and 97%, respectively (P = 0.55). But earlier study from India showed 86.5% in spot 1 in 1998 and 90.5% in 1999, 98.5% in the morning specimen in 1998 and 96.4% in 1999, and in spot 2, 83% in 1998 and 85.9% in 1999 (P < 0.001 in both years) [17]. The study from Tanzania showed the positivity rate for the first smear of 94.2% with incremental rate of 5.2 and 0.6% for the second and third smears, respectively [13]. Similar findings are reported from study done in Rwanda [15]: 92% detected in the first spot exam.

In addition, 93.9% of smear positive patients in this study had positive smears in all the three smears done regardless of smear grading, as compared to only 79% in the Indian study above. The difference may be due to quality of the sputum sample and set up under which the studies were done.

Examining multiple smears did not significantly increase the sputum smear positivity in Asella hospital. Much resource (time, money, laboratory chemicals, and human labor) was lost by dealing with multiple smears without getting additional advantage of more smear positives. In addition, it lead to delay in starting anti-TB medications.

The morning sputum sample showed a significant high-grade positivity. High-grade smear positivity is associated with lower smear conversion rate at the end of intensive phase of anti-TB treatment, and even after extending it for 1 month; therefore they are likely to remain infectious with high disease transmission [20, 21] and have less TB treatment outcome [22]. Therefore, examining the morning sputum sample alone can detect almost all patients likely to have smear-positive TB as well as more patients that are likely to have highly infectious TB. But this requires 2 days to stay in the health institution likely leading to some patient loss. For those laboratories with external quality assurance, the current WHO recommendation is to switch to same-day diagnosis using spot–spot smears, [23, 24] This is also supported by a more recent systematic review and meta analysis [25].

No gender difference in smear positivity in this study, but there is significant age difference; the highest smear positivity is seen in the 15–25 age group. This is similar to the study done in Rwanda [14]. Ethiopia has a broad-based- pyramid population pattern. Therefore, much of the young and productive age group that can play significant role in the country’s economic development, are being affected heavily.

## Limitations

As the study is retrospective, it may affect the findings. It may not be possible to generalize and apply the findings to other hospitals with fewer resources in Ethiopia.

## Additional file

**Additional file 1.** Additional figures.

## Abbreviations

AFB: acid fast bacilli
FDRE: Federal Democratic Republic of Ethiopia
HIV: human immunodeficiency virus
IUALD: International Union against Lung Diseases
PTB: pulmonary tuberculosis
SPSS: Statistical Package for Social Sciences
TB: tuberculosis
WHO: World Health Organization
