# Supplementary material for: The value of one versus three sputum smear examinations for diagnosis of pulmonary tuberculosis in Asella hospital, South-East Ethiopia
Source: BMC Res Notes. 2017 Sep 6;10:455. doi: 10.1186/s13104-017-2797-0 (PMC5588549; doi:10.1186/s13104-017-2797-0)
Supplement: Supplementary file 1 — Additional file 1. Additional figures. [file 13104_2017_2797_MOESM1_ESM.doc]

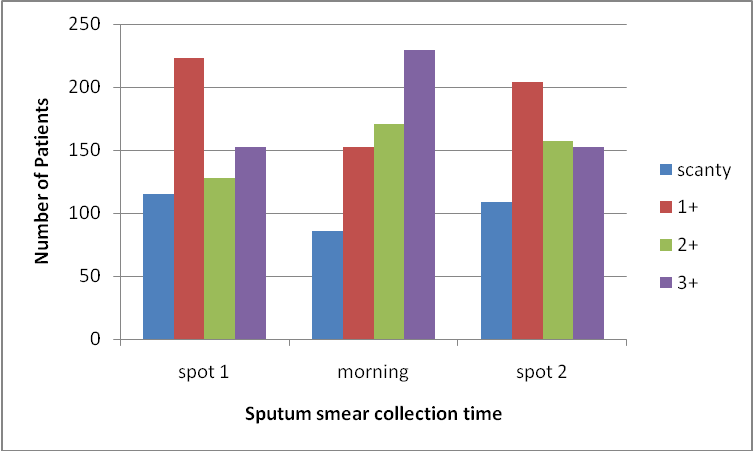


Figure 1: Sputum smear grading against time of collection

*Spot 1 and spot 2 smears have similar sensitivity of detecting sputum smears, especially 1+. Most of the high grade smears (3+) are detected in the morning sputum*.


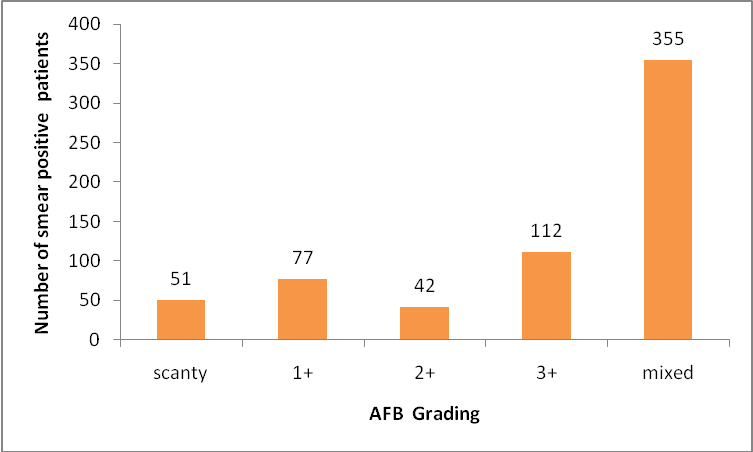


Figure 2: Sputum smear grading of smear positive patients

*Most of the patients had mixed sputum smear grading (55.7%). The rest of them had similar smear grades in all the three samples: all samples scanty in 51, all samples 1+ in 77, all 2+ in 42 and all 3+ in 112.*
